# Treatment of Symptomatic Lower Pole Stones of a Kidney with Partial Nephrectomy Using Micropercutaneous Nephrolithotomy Technique

**DOI:** 10.1155/2015/456714

**Published:** 2015-03-31

**Authors:** Tuna Karatag, Ibrahim Buldu, Mehmet Kaynar, Hakan Taskapu, Erdem Tekinarslan, Mustafa Okan Istanbulluoglu

**Affiliations:** ^1^Department of Urology, Mevlana University, 42040 Konya, Turkey; ^2^Department of Urology, Selçuk University, Konya, Turkey; ^3^Department of Urology, Necmettin Erbakan University, Konya, Turkey; ^4^Department of Urology, Konya Education and Research Hospital, Konya, Turkey

## Abstract

We present the treatment of lower pole stones of a 62-year-old male patient with a history of open partial nephrectomy due to renal angiomyolipoma and renal stones. He was successfully treated with micropercutaneous nephrolithotomy technique under spinal anesthesia in spite of fibrotic and scar tissue due to previous open surgery. The patient was stone-free and was discharged after a 24-hour hospitalization period. There is not any published report of micropercutaneous nephrolithotomy in a partial nephrectomized kidney before. In this report, we suggest that microperc technique may be considered for challenging conditions in case of failed retrograde intrarenal surgery.

## 1. Introduction 

Following introduction of optic puncture system in renal stone treatment in 2011 [1], micropercutaneuos nephrolithotomy (microperc) procedures have become a choice for the treatment of kidney stones in size of 10–20 mm [2, 3]. Although the initial reports have suggested that microperc technique was feasible, safe, and efficacious for the small and moderate size kidney stones [3], there are only a few reports for the complicated cases. We aimed to report the treatment of lower pole stones (LPSs) of a kidney with fibrotic and scar tissue due to open partial nephrectomy using a tubeless microperc technique.

## 2. Case Presentation and Method

**Figure 1:**
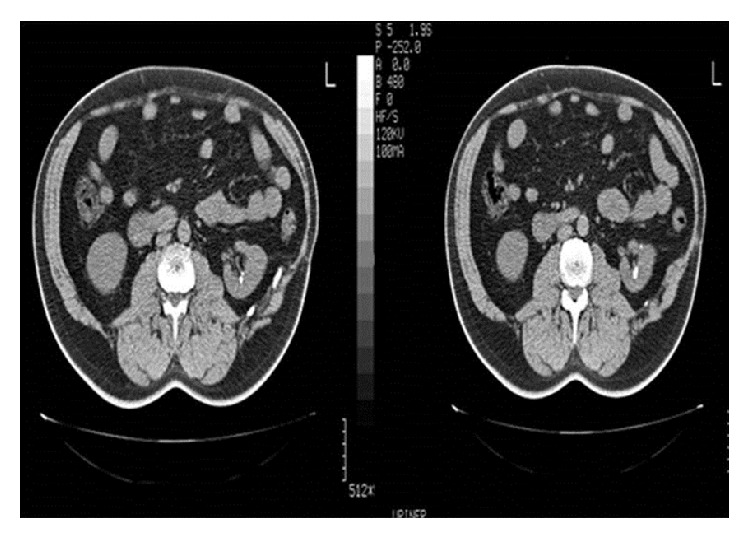
Preoperative CT imaging showing left kidney stone.

**Figure 2:**
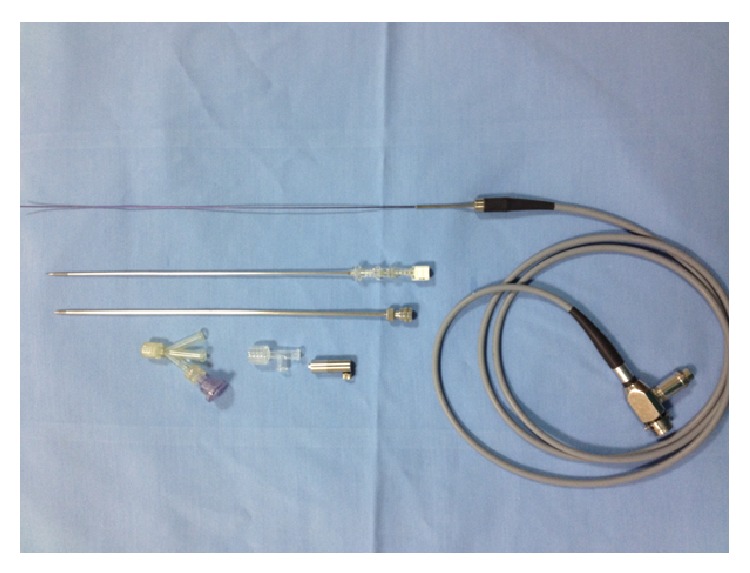
Needle, flexible microfiber optic, and three-way adaptor.

A 62-year-old male patient was referred to our clinic and presented with complaints of pain in the left lumbar region and recurrent urinary tract infections. He had also a previous history of open partial nephrectomy due to a four-centimeter left renal angiomyolipoma before presentation. Computer tomography (CT) revealed the presence of three LPSs where the largest one was 10 mm and identified the condition of the surrounding organs (Figure 1). The patient underwent microperc procedure under spinal anesthesia. After inserting a 5 Fr open-end ureteral catheter into the left ureter by cystoscopy, the pelvicalyceal system was filled with diluted contrast agent injected through the ureter. Then, a 0.6 mm diameter flexible microfiber optic within a needle of 1.6 mm diameter (4.85 Fr/16 gauge) and the modified three-part needle (PolyDiagnost, Pfaffenhofen, Germany) were used to access the stone in the collective system in prone position with fluoroscopy guidance from the posterior axillary line. We placed an 8 Fr sheath into the kidney with the guidance of 0.038 inch PTFE hydrophilic sensor guidewire. We were allowed to reach all stones through direct visualization in spite of scarring and fibrosis due to previous open surgery. A three-way adaptor for irrigation, laser, and flexible microfiber optic insertion was assembled to the proximal end of the needle (Figure 2). Images gained by the microfiber optic system were monitored via multijoint arm using standard endoscopic camera system and xenon light source. Holmium: YAG laser 200 *μ*m fibers, 8 Hz frequency and 0.7 joule energy (Quanta System Laser Litho, Italy), were used for lithotripsy. We removed the sheath at the end of the operation and kept the open-end catheter in place.

## 3. Results

**Figure 3:**
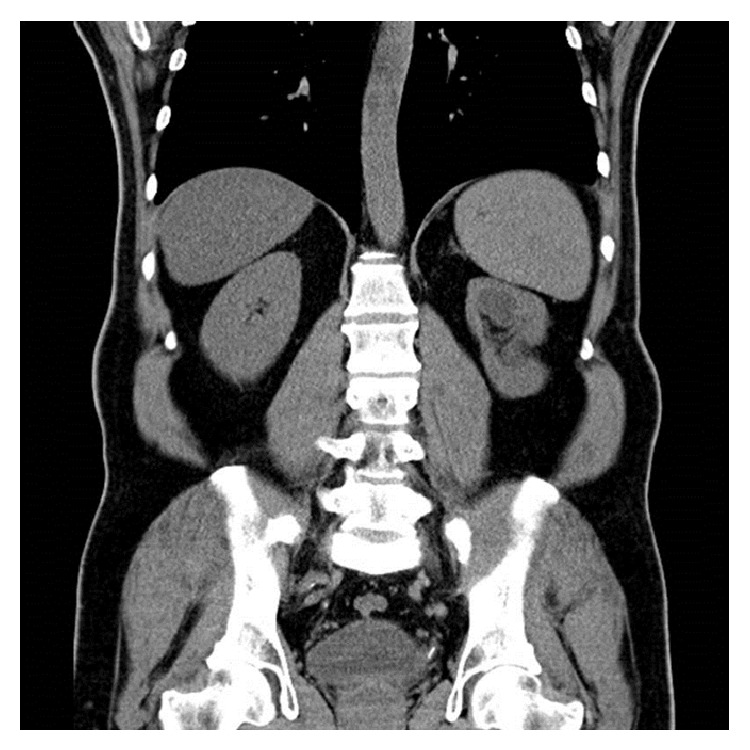
Postoperative CT image at the first month.

Total operation time was 30 minutes and fluoroscopy time was 25 seconds. While a great hemorrhage did not occur during the operation, there were not any intraoperative or postoperative complications. Hemoglobin drop was 0.5 mg/dL. The following day, the ureteral catheter was removed after a duration time of 18 hours. There were stone fragments in size of 1 to 2 mm on postoperative day one's plain image. The patient was discharged after a total of 24 hours' hospitalization period. We achieved a stone-free status at first month control after spontaneous passage of fragments (Figure 3).

## 4. Discussion

Since percutaneous nephrolithotomy technique was first established in 1976 by Fernstrom and Johansson for the management of upper urinary tract stones [4], technological advancements and improvements in instruments have further contributed to this procedure becoming currently primary treatment option for most of the large and/or multiple renal stones [5, 6]. The main aim of all these refinements is to contribute to further lowering the morbidity associated with this procedure.

Recently, Desai et al. presented the tubeless microperc technique for the renal stone treatment that was achieved using 4.85 Fr instruments under direct visualization of renal access [2]. The author reported that this technique was feasible, safe, and efficient in small size renal stones as a conclusion in the first microperc series consisting of 10 patients.

In another current series, Karatag et al. reported their initial experience with microperc in the treatment of patients with moderately sized renal calculi [3]. It was also highlighted that microperc procedure was safe and efficacious for kidney stones in size of 10 to 20 mm. Moreover, in a comparative study, it was also suggested that microperc procedure was reliable and feasible even under spinal anesthesia and might be an alternative to SWL [7].

Regarding our present case report, we aimed to highlight that microperc technique may be considered for medium sized lower pole stones in challenging cases such as having a history of previous open partial nephrectomy. According to our knowledge, this present case is the first report about the reliability of microperc procedure for the treatment of lower pole stones in a partial nephrectomized kidney. Scarring and fibrosis due to previous open surgery might prolong the operative time; however, total operative time was only 30 minutes. Meanwhile, using a larger sheath and nephroscope in standard PNL might increase the severity of hemorrhage and the duration of operation. Furthermore, prolonged operative time might be responsible for the development of complications such as infection, embolization, and anesthesia related complications. Moreover, instrument size might affect the postoperative pain and rapid recovery of tissues. Another advantage of microperc with optic puncture system is that all layers are passed from the skin until the lower collective system via fiber optic displayed images on the monitor without dilatation in a single step. Owing to this facility, safety access can be applied without harming the surrounding organs and without causing major bleeding.

However, extracorporeal shock wave lithotripsy (SWL) modality may have some limitations for the present case. Larger sizes of LPSs and anatomic abnormalities might decrease the success rates for SWL approach. We can also consider an approach of retrograde intrarenal surgery with flexible instruments for our present case. Unfortunately, flexible instruments were not available in our clinic in that period. Thereby, we performed microperc surgery for the present case.

## 5. Conclusions

We suggest that microperc technique is reliable and efficacious for the treatment of medium sized LPSs of challenging cases as mentioned in our present case. Microperc may be considered for such conditions in the absence of flexible ureterorenoscopy.

## Abbreviations

SWL: Shock wave lithotripsy
PNL: Percutaneous nephrolithotomy
RIRS: Retrograde intrarenal surgery
KUB: Kidney-ureter-bladder.
